# Supplementary material for: The AAA ATPase Vps4 binds ESCRT-III substrates through a repeating array of dipeptide-binding pockets
Source: eLife. 2017 Nov 22;6:e31324. doi: 10.7554/eLife.31324 (PMC5716660; doi:10.7554/eLife.31324)
Supplement: Supplementary file 1. — This is for the parts of the model that were defined in charge density at a resolution that justified refinement (Subunits A-E, nucleotides, ESCRT-III peptide). Data in Table 1 are based on this report. [file elife-31324-supp1.docx]

9/12/2017 Analysis output: all-atom contacts and geometry for Vps4_model_subunitA-EH.pdb - MolProbity

**Analysis output: all- atom contacts and geometry for Vps4_model_subunitA- EH.pdb**


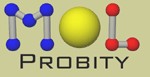

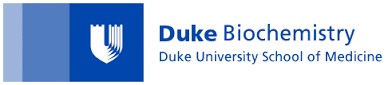


# Summary statistics

| All-Atom Contacts | Clashscore, all atoms: | 5.02 | | 100th percentile* (N=41, 2.95Å - 9999Å) |
| --- | --- | --- | --- | --- |
|  | Clashscore is the number of serious steric overlaps (> 0.4 Å) per 1000 atoms. | | | |
| Protein Geometry | Poor rotamers | 0 | 0.00% | Goal: <0.3% |
|  | Favored rotamers | 1161 | 97.81% | Goal: >98% |
|  | Ramachandran outliers | 0 | 0.00% | Goal: <0.05% |
|  | Ramachandran favored | 1240 | 89.53% | Goal: >98% |
|  | MolProbity score^ | 1.83 | | 100th percentile* (N=1544, 3.20Å ± 0.25Å) |
|  | Cβ deviations >0.25Å | 0 | 0.00% | Goal: 0 |
|  | Bad bonds: | 1 / 11113 | 0.01% | Goal: 0% |
|  | Bad angles: | 27 / 15002 | 0.18% | Goal: <0.1% |

In the two column results, the left column gives the raw count, right column gives the percentage.

* 100th percentile is the best among structures of comparable resolution; 0th percentile is the worst. For clashscore the comparative set of structures was selected in 2004, for MolProbity score in 2006.

^ MolProbity score combines the clashscore, rotamer, and Ramachandran evaluations into a single score, normalized to be on the same scale as X-ray resolution.

# Multi-criterion visualizations


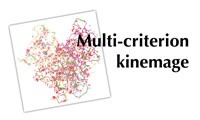

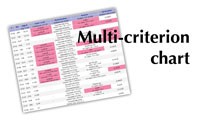


View in KiNG | [Download](http://molprobity.biochem.duke.edu/data/lt945sk3h152j2gdbjsg5g3ql4/kinemages/Vps4_model_subunitA-EH-multi.kin.gz) (4.4 Mb) View (1.4 Mb)

# Single-criterion visualizations

**Clash list** (4.7 Kb): View

**Ramachandran plot kinemage** (493 Kb): View in KiNG | [Download](http://molprobity.biochem.duke.edu/data/lt945sk3h152j2gdbjsg5g3ql4/kinemages/Vps4_model_subunitA-EH-rama.kin) **Ramachandran plot PDF** (1.7 Mb): [View](http://molprobity.biochem.duke.edu/data/lt945sk3h152j2gdbjsg5g3ql4/charts/Vps4_model_subunitA-EH-rama.pdf)

**Cβ deviation scatter plot** (95 Kb): View in KiNG | [Download](http://molprobity.biochem.duke.edu/data/lt945sk3h152j2gdbjsg5g3ql4/kinemages/Vps4_model_subunitA-EH-cbetadev.kin)


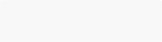


Continue >

About [MolProbity](http://molprobity.biochem.duke.edu/help/about.html) | Website for [the Richardson Lab](http://kinemage.biochem.duke.edu/) | Using ecloud x-H | Internal reference 4.4

[http://molprobity.biochem.duke.edu/index.php?MolProbSID=lt945sk3h152j2gdbjsg5g3ql4&eventID=98](http://molprobity.biochem.duke.edu/index.php?MolProbSID=lt945sk3h152j2gdbjsg5g3ql4&amp;eventID=98) 1/1
